# KRT5^+^/p63^+^ Stem Cells Undergo Senescence in the Human Lung with Pathological Aging

**DOI:** 10.14336/AD.2022.1128

**Published:** 2023-06-01

**Authors:** Manuel Moreno-Valladares, Veronica Moncho-Amor, Tulio M Silva, Juan P Garcés, María Álvarez-Satta, Ander Matheu

**Affiliations:** ^1^Biodonostia Health Research Institute, Group of Cellular Oncology, San Sebastian, Spain; ^2^Donostia University Hospital, Pathology Department, San Sebastian, Spain; ^3^CIBER of Frailty and Healthy Aging (CIBERfes), Carlos III Institute, Madrid, Spain; ^4^IKERBASQUE, Basque Foundation for Science, Bilbao, Spain

**Keywords:** Human lung, aging, age-related respiratory disease, stem cells, KRT5, p63, cellular senescence

## Abstract

Adult lungs present high cellular plasticity against stress and injury, mobilizing stem/progenitor populations from conducting airways to maintain tissue homeostasis and gas exchange in alveolar spaces. With aging, pulmonary functional and structural deterioration occurs, mainly in pathological conditions, which is associated with impaired stem cell activity and increased senescence in mice. However, the impact of these processes underlying lung physiopathology in relation to aging has not been explored in humans. In this work, we analyzed stem cell (SOX2, p63, KRT5), senescence (p16^INK4A^, p21^CIP^, Lamin B1) and proliferative (Ki67) markers in lung samples from young and aged individuals, with and without pulmonary pathology. We identified a reduction in SOX2^+^ cells but not p63^+^ and KRT5^+^ basal cells in small airways with aging. In alveoli, we revealed the presence of triple SOX2^+^, p63^+^ and KRT5^+^ cells specifically in aged individuals diagnosed with pulmonary pathologies. Notably, p63^+^ and KRT5^+^ basal stem cells displayed colocalization with p16^INK4A^ and p21^CIP^, as well as with low Lamin B1 staining in alveoli. Further studies revealed that senescence and proliferation markers were mutually exclusive in stem cells with a higher percentage colocalizing with senescence markers. These results provide new evidence of the activity of p63^+^/KRT5^+^ stem cells on human lung regeneration and point out that regeneration machinery in human lung is activated under stress due to aging, but fails to repair in pathological cases, as stem cells would likely become senescent.

## INTRODUCTION

Human lungs are mainly divided in two distinct areas: The conducting airways, including the trachea, bronchi and bronchioles, and the gas exchange region or alveolar spaces. The lung is an organ that remains largely quiescent with relatively slow cell turnover. However, it presents high cellular plasticity against stress and injury, mobilizing different stem/progenitor populations (hereinafter referred to as stem cells) to regenerate the damaged tissue [1, 2]. In humans, lung stem cells are distributed along the respiratory epithelium and include airway basal cells (the major stem cell population in human airways), bronchiolar secretory cells (known as club cells), and alveolar type II cells [1, 2]. However, it is not clear which subpopulations proliferate and/or mobilize to repair the human lung in the context of physiology and pathology.

Most of the knowledge about lung regeneration comes from studies in mouse models where stem cells located in distal small airways are the main players in lung homeostasis and regeneration. Thus, a population of immature, distal stem cells positive for keratin 5 (KRT5) and tumor protein p63 (p63) expands and mobilizes to the alveolar region after influenza infection forming dysplastic KRT5^+^/p63^+^ pods able to regenerate lung parenchyma [3-5]. Additional studies have shown that the predominant source of nascent KRT5^+^/p63^+^ pods in mouse small airways and alveoli are proximal airway-resident SOX2^+^/SCGB1A1^-^/KRT5^-^ progenitor cells. Indeed, the regenerating response initiates in the proximal airways and KRT5^+^ cells then migrate and repopulate the damaged alveolar region [6]. Remarkably, these KRT5^+^ clusters were also observed in fibrotic human lungs [4] and human distal airway stem cells are able to form these alveoli-like structures *in vitro* [3].

Aging consists of a progressive loss of tissue integrity and functional reserve with age, together with an impaired regenerative response to injury, which affects all organs and systems. In the case of lungs, their functional decline is manifested as impaired pulmonary gas exchange, reduced lung elastic recoil and disturbed compliance, which progressively lead to a lower respiratory capacity and higher susceptibility to develop respiratory diseases [7]. Histologically, old individuals display enlarged alveoli, thickening of alveolar walls, distal duct ectasia, and reduced density of lung capillaries, among other features [8]. At molecular level, higher levels of pro-inflammatory cytokines such as interleukin 1 beta, interleukin 6 and tumor necrosis factor alpha, a reduced immune response, and structural alterations in extracellular matrix haven been reported in older lungs [9].

Changes in stem cell dynamics are thought to contribute to the age-related impairment of pulmonary regeneration and disease development, although no evidence is available for humans. Thus, the number of tracheal lung stem cells is reduced with normal aging in mice together with a decline in density and proliferative activity of distal lung stem cells (bronchiolar club and alveolar type II cells) [10, 11]. Moreover, old mice have less cellular density and lower proportion of KRT5^+^/p63^+^ basal cells in the tracheal epithelium, suggesting that self-renewal and reparative abilities of lung proximal stem cells decline with age [10, 12, 13]. In addition, the expression of transcriptionally active isoforms of p63 decreases in an age-dependent manner in lungs from mice and rhesus monkeys [14]. Cellular senescence also represents a critical process in terms of regenerative capacity and aging [15]. In this sense, an increased expression of the senescence markers p16^INK4A^, retinoblastoma and heterochromatin protein macro H2A has been reported in the lungs from individuals aged >50, mainly in the vasculature but also in lung parenchyma [16, 17]. Moreover, the role of cellular senescence in the physiopathology of chronic respiratory diseases such as chronic obstructive pulmonary disease (COPD) and idiopathic pulmonary fibrosis (IPF), for which increasing age represents a major risk factor, is increasingly recognized [18, 19]. Thus, COPD resembles a process of accelerated lung aging, where a high proportion of senescent alveolar type II cells and pulmonary endothelial cells, together with a pro-inflammatory senescence-associated secretory phenotype (SASP) environment, contribute to the structural and functional decline of COPD lungs [20-22]. Cellular senescence is also involved in IPF pathogenesis, a devastating disease characterized by progressive pulmonary fibrosis that compromises lung function. Thus, an accumulation of the senescence markers p16^INK4A^, p21^CIP^, senescence-associated β-galactosidase activity, and SASP factors in alveolar epithelial cells and fibroblasts has been described in human IPF lung tissue [23-25]. In addition, increased p16^INK4A^ expression correlates with the severity of disease in patients [26, 27].

So far, there are no studies that address the expression and clinical relevance of stem cell dynamics and cellular senescence in human lung homeostasis with physiological and pathological aging. In this work, we analyzed the presence and distribution of stem cell (SOX2, p63 and KRT5), senescence (p16^INK4A^, p21^CIP^ and Lamin B1) and proliferation (Ki67) markers, as well as markers of regeneration in lung samples from young and aged individuals, with and without pulmonary pathology.

## MATERIAL AND METHODS

### Samples

Human lung samples (n=24) were mainly collected from autopsies conducted at Donostia University Hospital (Spain) and include three biopsies from young patients. Postmortem interval was limited to 12 hours due to its effects on pulmonary proteins. Lungs were kept in a fixative solution (4% paraformaldehyde) for a period not less than 24 h. Samples were divided in three groups: “young” (n=6; individuals ranged from 17-43 years old), “aged” (n=10; individuals aged 61-88 years old) and “lung pathology” (n=8; individuals between 57-85 years old). Inclusion criteria for selection of “young” and “aged” patients included the absence of clinical diagnosis of pulmonary disease as well as the lack of lung injury in the regions analyzed. Patients with lung cancer were excluded. Patient's information is compiled in Table 1.

**Table 1:** Clinical features of patients enrolled in this study.

GROUP	AGE	SEX	DIAGNOSIS/CAUSE OF DEATH	STRUCTURAL INJURY
Young	36	F	Sepsis	-
Young	35	M	Sepsis	-
Young	43	M	Hepatic failure	-
Young	17	M	Spontaneous pneumothorax	-
Young	18	M	Spontaneous pneumothorax	-
Young	19	M	Spontaneous pneumothorax	-
Aged	63	F	Subarachnoid hemorrhage	-
Aged	61	M	Ruptured abdominal aneurysm	-
Aged	78	F	Hypovolemic shock	-
Aged	84	F	Pulmonary thromboembolism	-
Aged	83	F	Sepsis	-
Aged	83	F	Lymphoma	-
Aged	76	F	Colonic ulcer	-
Aged	88	F	Melanoma brain metastasis	-
Aged	83	M	Amyotrophic lateral sclerosis	-
Aged	72	F	Encephalitis	-
Lung pathology	72	M	Pulmonary fibrosis	Dysplastic airway-like structures
Lung pathology	60	M	Diffuse alveolar damage	No dysplastic airway-like structures
Lung pathology	85	F	Chronic obstructive pulmonary disease	Dysplastic airway-like structures
Lung pathology	77	M	Chronic obstructive pulmonary disease	No dysplastic airway-like structures
Lung pathology	76	F	Diffuse interstitial lung disease	Dysplastic airway-like structures
Lung pathology	66	F	Diffuse alveolar damage	Dysplastic airway-like structures
Lung pathology	75	F	Ischemic stroke, pneumoconiosis	No dysplastic airway-like structures
Lung pathology	57	M	Aspiration pneumonia	No dysplastic airway-like structures

Abbreviations: *F*-Female, *M*-Male. Age is shown in years.

This study was approved by the Clinical Research Ethics Committee of the Donostia University Hospital (AMM-MHP-2019-1) and adhered to the tenets of the Declaration of Helsinki by the World Medical Association regarding human experimentation.

### Immunohistochemistry of lung slides

Tissue slides for immunohistochemistry (IHC) were obtained as follows: Briefly, lung lobes from autopsies were extracted, fixed in formalin, paraffin-embedded and subsequently sectioned in 4 μm sections with a Leica rotary microtome. Sections from peripheral and central regions of right and left lung lobes were obtained for further analysis. In the case of lung samples coming from biopsies, the same procedure was followed with the specific sample obtained. IHC was performed following the manufacturer's instructions on the Roche Ventana Benchmark ULTRA System with ethylenediaminetetraacetic acid (EDTA) pH 8.5 antigen retrieval. The following primary antibodies were used: SOX2 (Roche Ventana, cat number: 760-4621), p63 (Roche Ventana, cat number: 790-4509), KRT5 (Roche Ventana, cat number: 790-4554), TTF-1 (Roche Ventana, cat number: 790-4756), p16^INK4A^ (Roche Ventana, cat number: 805-4713), p21^CIP^ (Roche Ventana, cat number: 760-4453), Lamin B1 (Cell Signaling Technology, cat number: 12586S; dilution 1:300), Ki67 (Roche Ventana, cat number: 790-4286) and KRT14 (Roche Ventana, cat number: 760-4805). Hematoxylin-eosin was performed using standard procedures. Sections were visualized and scanned with Virtuoso v.5.6.1 software (Ventana Medical Systems, Roche).

### Immunofluorescence of lung slides

Immunofluorescence (IF) was performed in formalin-fixed lung samples. Paraffin-embedded tissue sections were deparaffinized in xylene, rehydrated in a series of graded alcohols, and then heated in citrate buffer during 1 h for antigen retrieval. Tissues were permeabilized with 0.5% Triton X-100 (PBS-T; Sigma-Aldrich) and blocked for 1 h with 1% bovine serum albumin and 5% goat-serum (Sigma-Aldrich) in PBS-T. Sections were incubated at 4ºC overnight with the following primary antibodies: SOX2 (Neuromics, cat number: GT15098; dilution 1:300), p63 (Abcam, cat number: ab124762; 1:300), KRT5 (Roche Ventana, cat number: 790-4554), p16^INK4A^ (Roche Ventana, cat number: 805-4713), p21^CIP^ (Thermo Fisher Scientific, cat number: MA5-14949; 1:100), Lamin B1 (Cell Signaling Technology, cat number: 12586S; 1:300), Ki67 (Abcam, cat number: ab15580 and Thermo Fisher Scientific, cat number: 14-5698-82; 1:300 and 1:100, respectively), KRT14 (Roche, cat number: 760-4805) and KRT8 (Abcam, cat number: ab234348; 1:100). Sections were washed in PBS-T and then incubated for 1 h at room temperature with the corresponding anti-rat, anti-goat or anti-rabbit secondary antibody conjugated to Alexa Fluor 488, 555, 594 or 647 (Thermo Fisher Scientific) in 10% blocking solution (1:600) with 1 μM 4’,6-diamino-2-phenylindole (DAPI). Finally, sections were washed with PBS-T and mounted using ProLong™ Gold Antifade Mountant (Invitrogen™). Images were acquired using a Zeiss LSM 900 confocal microscope. Settings were established during the initial acquisition.

### Cell quantification

For IHC, the quantification of positive cells for the different markers was manually performed from previously scanned images. Two pulmonary regions were checked: the airway region (“conducting zone”, which includes segmental bronchi and bronchioles), and the gas exchange portion (“exchange zone”, composed of alveolar sacs and alveoli). For quantification, two independent fields of each region (conducting zone or exchange zone) were inspected in each patient at 200× magnification. In each field, all positive cells for each marker relative to total nuclei were considered.

In the case of IF, three different fields were chosen on different sections (conducting zone or exchange zone). Within these, positive cells for the different markers and combinations analyzed were counted manually and blindly. The Fiji software was used to pseudocolor the different channels in unprocessed micrographs. The QuPath software [28] was also used for image analysis. Briefly, areas of interest were defined as respiratory and alveolar epithelium. Within the areas of interest, the centre of cell nuclei was then identified as maxima in a filtered DAPI image. Nuclear boundaries were assigned by a propagation algorithm, and then expanded by ~1 µm to define sampling areas. The following data were then recorded: (i) average pixel intensities for each data channel over each sampling area, representing one cell; (ii) the size of the sampling areas; and/or (iii) the number of positive cells for each data channel over each sampling area.

### Statistical analysis

**Figure 1.**
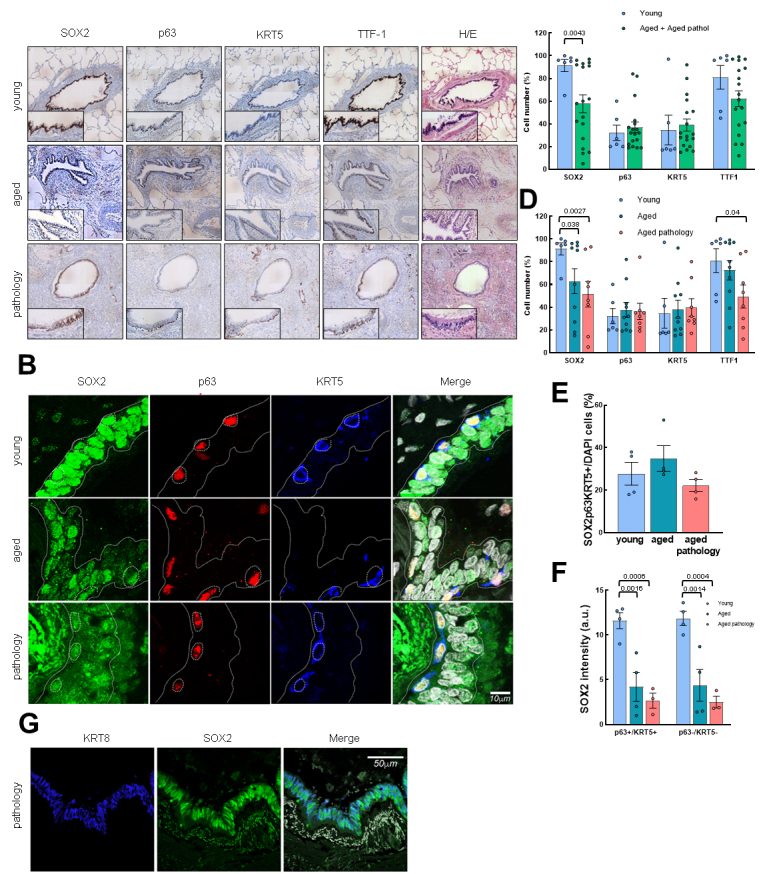
p63, KRT5 and SOX2 expression defines distinct cell populations in conducting airway epithelium. (A) Representative IHC pictures (4x) of SOX2, p63, KRT5 and TTF-1 markers in the three groups (rows). Zoomed areas (20x) for each picture are in black frames. Hematoxylin-eosin staining is also shown. (B) Representative IF staining of SOX2, p63, KRT5 and merged pictures with DAPI counterstaining. Scale bar: 10 µm. (C) Quantification of cells positive for SOX2, p63, KRT5 and TTF-1 markers by IHC in young (n=6) and grouped aged (n=18) patients. (D) Quantification dividing aged cases in no lung pathology (n=10) and with pulmonary pathology (n=8). Data are shown as percentage of marked cells relative to total nuclei. (E) Quantification of triple positive cells for SOX2, p63 and KRT5 markers relative to total nuclei in the three groups (young: n=4; aged: n=4; aged with pulmonary pathology: n=4). (F) Quantification of SOX2 fluorescence intensity in the three groups (young: n=4; aged: n=4; aged with pulmonary pathology: n=3). Left, quantification in cells p63/KRT5 double positive; right, in p63/KRT5 double negative. (G) Representative IF pictures of SOX2 and KRT8 staining in aged pathology patients. Merged pictures and DAPI counterstaining are also shown. Scale bar: 50 µm. Data represent average ± SEM. p-value when statistical significance was reached, is shown. *a.u*.: arbitrary units.

**Figure 2.**
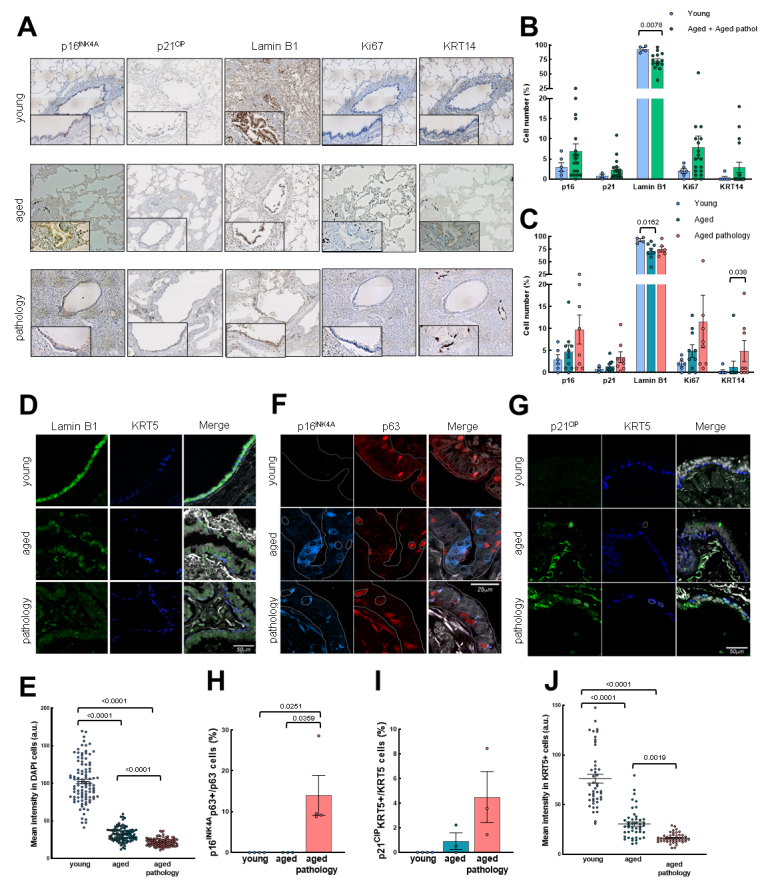
Senescent cells in conducting airway epithelium and colocalization with basal cell markers. (A) Representative IHC pictures (4x) of p16^INK4A^, p21^CIP^, Lamin B1, Ki67 and KRT14 markers in the three groups (rows). Zoomed areas (20x) for each picture are in black frames. (B) Quantification of cells positive for p16^INK4A^, p21^CIP^, Lamin B1, Ki67 and KRT14 markers by IHC in young (n≥3) and grouped aged (n≥14) patients. (C) Quantification as performed in B by dividing patients in the three original groups (young: n≥3; aged: n≥8; aged with pulmonary pathology: n≥6). Data are shown as percentage of marked cells relative to total nuclei for B and C. (D-E) Representative IF of Lamin B1, KRT5 and quantification of mean nuclei fluorescent intensity per cell of Lamin B1 (young: n=3; aged: n=3; aged with pulmonary diseases: n=4). (F-G) Representative IF of p16^INK4A^/p63 and p21^CIP^/KRT5 with merged pictures and DAPI counterstaining in the three groups (rows). Scale bar: 25 and 50 µm, respectively. (H, I) Quantification of p16^INK4A^/p63 and p21^CIP^/KRT5 double positive cells relative to p63 and KRT5 positive cells, respectively (young: n=4; aged: n=3; aged with pulmonary diseases: n≥3). (J) Quantification of mean nuclei fluorescent intensity of Lamin B1 per KRT5 positive cell (young: n=3; aged: n=3; aged with pulmonary diseases: n=4). Data represent average ± SEM. p-value when statistical significance was reached, is shown.

**Figure 3.**
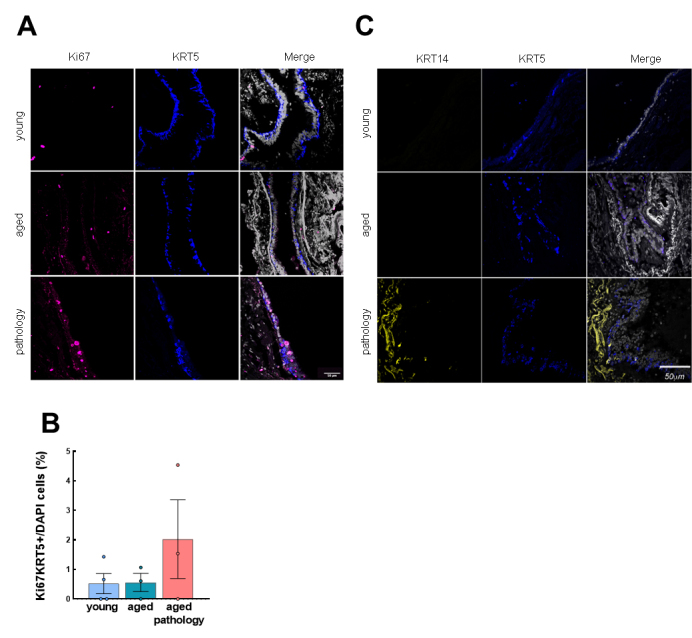
Characterization of KRT5^+^ basal cells in the conducting airway epithelium. (A) Representative IF pictures of Ki67/KRT5 and merged pictures and DAPI counterstaining in the three groups (rows). Scale bar: 50 µm. (B) Quantification of double positive cells for Ki67 and KRT5 relative to total nuclei (young: n=4; aged: n=3; aged with pulmonary pathology: n=3). (C) Representative IF pictures of KRT14/KRT5 and merged pictures and DAPI counterstaining in the three groups (rows). Scale bar: 50 µm. Data represent average ± SEM.

**Figure 4.**
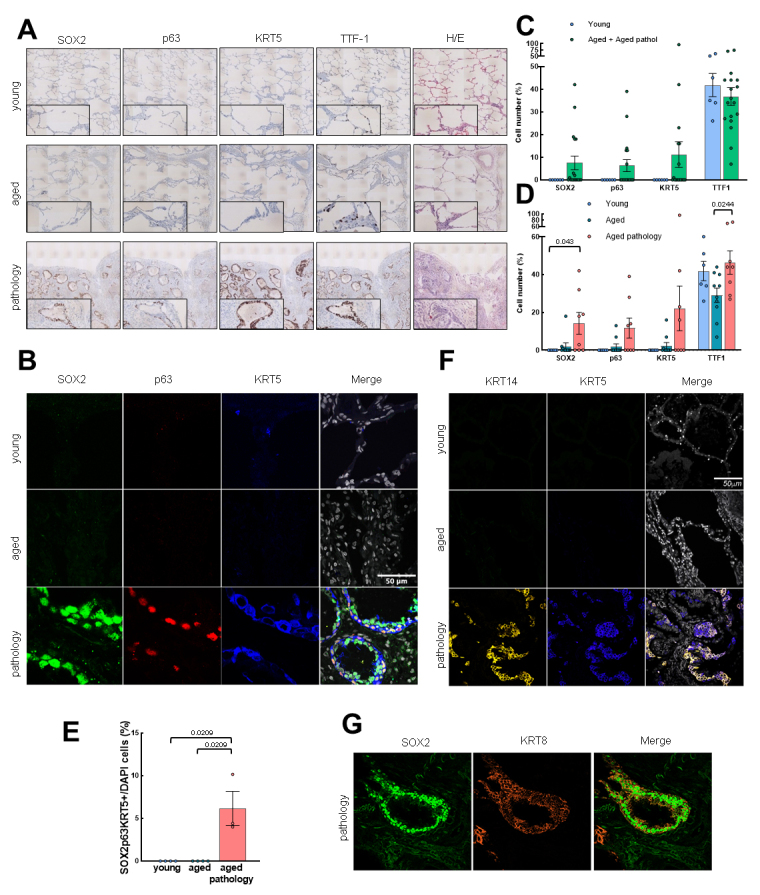
Basal cell marker expression increases with pathological aging in the exchange zone. (A) Representative IHC pictures (4x) of SOX2, p63, KRT5 and TTF-1 in the three groups (rows). Zoomed areas (20x) for each picture are in black frames. Hematoxylin-eosin staining is also shown. (B) Representative IF pictures of SOX2, p63, KRT5 and merged pictures and DAPI counterstaining in the three groups (rows). Scale bar: 50 µm. (C) Quantification of cells positive for SOX2, p63, KRT5 and TTF-1 by IHC in young (n=6) and grouped aged (n=18) patients. (D) Quantification in the three groups (young: n=6; aged: n=10; aged with pulmonary pathology: n=8). Data are shown as percentage of marked cells relative to total nuclei. (E) Quantification of positive cells for SOX2, p63 and KRT5 relative to total nuclei in the three groups (young: n=4; aged: n=4; aged with pulmonary diseases: n=3). (F) Representative IF pictures of KRT14, KRT5 and merged pictures with DAPI counterstaining in the three groups (rows). Scale bar: 50 µm. (G) Representative IF pictures of SOX2, KRT8 and merged pictures with DAPI counterstaining in aged pathology cases. Scale bar: 50 µm. Data represent average ± SEM. p-value when statistical significance was reached, is shown.

**Figure 5.**
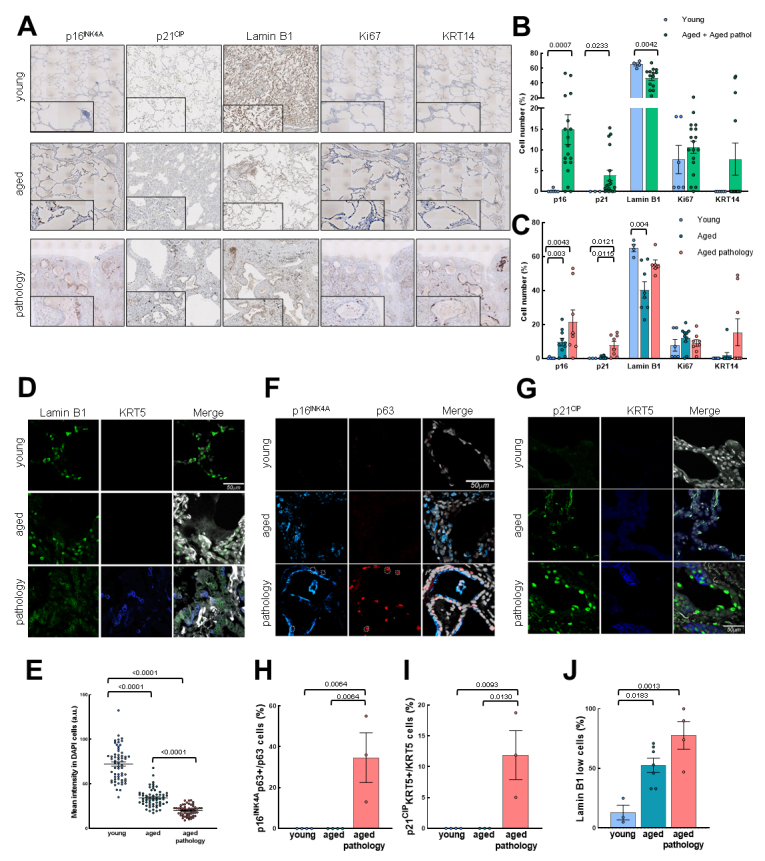
Colocalization of senescence markers and basal cells in the exchange zone with pathological aging. (A) Representative IHC pictures (4x) of p16^INK4A^, p21^CIP^, Lamin B1, Ki67 and KRT14 in the three groups (rows). Zoomed areas (20x) for each picture are in black frames. (B) Quantification of cells positive for p16^INK4A^, p21^CIP^, Lamin B1, Ki67 and KRT14 by IHC in young (n≥3) and grouped aged (n≥14). (C) Quantification in the three groups (young: n≥3); aged: n≥8; aged with pulmonary diseases: n≥6). Data are shown as percentage of marked cells relative to total nuclei. (D-E) Representative IF of Lamin B1, KRT5 and quantification of mean nuclei fluorescent intensity per cell of Lamin B1 (young: n=3; aged: n=3; aged with pulmonary pathology: n=4). (F-G). Representative IF of p16^INK4A^/p63 and p21^CIP^/KRT5 and merged pictures with DAPI counterstaining in the three groups (rows). Scale bar: 50 µm. (H, I) Quantification of p16^INK4A^/p63 and p21^CIP^/KRT5 double positive cells relative to p63 and KRT5 positive cells, respectively (young: n=4; aged: n≥3; aged with pulmonary diseases: n=3). (J) Percentage of cells with intensity of Lamin B1 staining below average of aged cases (young: n=3; aged: n=7; aged with pulmonary pathology: n=4). Data represent average ± SEM. p-value when statistical significance was reached, is shown.

**Figure 6.**
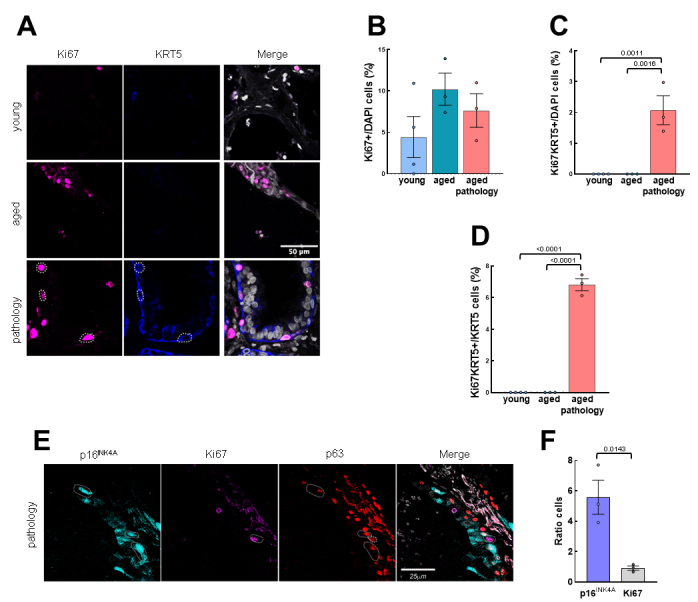
Proliferative basal cells are found in low number in aged patients with lung pathology in the exchange zone. (A) Representative IF pictures of Ki67/KRT5 and merged pictures with DAPI counterstaining in the three groups (rows). Scale bar: 50 µm. (B) Quantification of Ki67 positive cells relative to total nuclei (young: n=4; aged: n=3; aged with pulmonary diseases: n=3). (C) Quantification of positive cells for KRT5/Ki67 relative to total nuclei (young: n=4; aged: n=3; aged with pulmonary pathology: n=3). (D) Quantification of Ki67/KRT5 double positive cells relative to the KRT5 positive cells (young: n=4; aged: n=3; aged with pulmonary pathology: n=3). (E) Representative IF pictures of p16^INK4A^/Ki67/p63 and merged pictures with DAPI counterstaining in aged pathology cases with dysplastic airway-like structures. Scale bar: 25µm. (F) Ratio of senescent *vs.* proliferative cells in IF from E (n=3 per marker). Data represent average ± SEM. p-value when statistical significance was reached, is shown.

Statistical analyses were performed using Prism v.8.0c (GraphPad Software, USA). When comparing two groups of values where the distribution was non-parametric, Mann-Whitney test was performed (Fig. 1C-D, 2B-C, 4C-D, 5B-C). Student's t test was used in Fig. 6F. For multiple comparisons, analysis of variance (ANOVA) was implemented using Sidak’s multiple comparisons test in Fig.1E-F, Fig. 2E, 2H-J, Fig. 3B, Fig. 4E, Fig. 5E, 5H-J, Fig. 6B-D. Data are represented as average ± standard error of the mean (SEM). Regarding graphical representation, each dot represents one patient in all cases. Data were considered statistically significant when p-value<0.05.

### Data availability statement

The authors confirm that the data supporting the findings of this study are available within the article and/or its supplementary materials.

## RESULTS

### SOX2^+^ cells, but not p63^+^ and KRT5^+^ cells, decrease with aging in conducting airway epithelium

We first performed a histological analysis of lung samples from young and aged individuals. In young patients, this analysis revealed a normal lung architecture of bronchi, bronchioles and alveoli, with regular and thin walls, very low inflammation and normal organization of pseudostratified epithelium. In aged patients, the pulmonary architecture was well conserved, but more irregular and thicker airways and alveoli, with higher signs of inflammation, were observed (Fig. 1A). However, aged cases with lung pathology showed two kinds of damage of greater severity. On the one hand, exudative lesions where air spaces are mainly occupied by inflammatory infiltrates, edema and fibrin, but the alveolar architecture is well conserved. On the other hand, a structural injury characterized by a completely disrupted lung tissue structure, with absence of alveoli, widespread fibrosis and dysplastic airway-like structures with chronic inflammation focuses and mucus that replace normal alveoli.

Next, we analyzed the expression of SOX2, p63 and KRT5 stem cell markers by IHC and IF in small airways (“conducting zone”) in the three groups under study. First, we found that SOX2, p63 and KRT5^+^ cell localization was restricted to the airway epithelium in both young and aged groups (Fig. 1A, B and Supplementary Fig. 1A). IHC and IF also revealed a decrease in SOX2^+^ cells in the group of aged individuals (Fig. 1C and Supplementary Fig. 1B). A deeper analysis showed an average of more than 90% of positive cells in the young group compared to 62.9% and 51.4% in aged individuals and aged patients with pulmonary pathologies, respectively, by IHC (Fig. 1D). In contrast, the number of positive cells for p63 and KRT5 was lower, and the average was not altered with physiological or pathological aging (30-40% for the three sets) (Fig. 1D). In addition, cells positive for transcription termination factor 1 (TTF-1), a lineage marker of lung cells, were also reduced with aging, moving from about 80% of positive cells in young individuals to around 70% in aged ones, and 50% in aged patients with lung pathology (Fig. 1D). These results indicate that the number of SOX2^+^ cells, but not for p63 and KRT5 basal cell markers, is significantly reduced in small airways from aged individuals with physiological and pathological aging.

### Differential SOX2 expression defines distinct populations in conducting airway epithelium

To further characterize cell populations that may differentially express SOX2, p63 and KRT5 markers in the airways, we performed triple IF assays. We described that these three markers colocalized in basal cells (typical morphology of small, rounded nucleus with scarce and oval/rounded cytoplasm, distributed along the epithelial basal lamina) (Fig. 1B). Sporadically, some extended nuclei over the basal lamina were positive for p63, although with less intensity than well-characterized basal cells. IF also revealed that the number of SOX2^+^/p63^+^/KRT5^+^ basal cells remained similar for each group (Fig. 1E). Moreover, SOX2 was expressed in all epithelial cells that line the conducting airways, defining a second population characterized by the expression of SOX2 alone. In this case, SOX2 labelled differentiated, mature cylindrical cells with greater, oval nuclei that also integrate the airway epithelium. Furthermore, quantification analysis showed a reduction of SOX2 intensity in both basal cells (p63^+^/KRT5^+^) and the remaining epithelial cells (p63^-^/KRT5^-^) in aged patients without and with lung pathologies (Fig. 1F), with the latest expressing keratin 8 (KRT8) (Fig. 1G). Taken together, we identified two populations of epithelial cells in human conducting airways: one population of SOX2^+^/p63^+^/KRT5^+^ cells identified as the basal stem cell population, and other set of intermediate or differentiated cells that only express SOX2.

### Senescent cells are elevated in conducting airway epithelium

Next, we characterized the expression of the senescence markers p16^INK4A^, p21^CIP^ and Lamin B1, as well as the proliferative marker Ki67 (Fig. 2A). First, we detected a 2-fold increase in the percentage of p16^INK4A^ and p21^CIP^ positive cells with aging (Fig. 2B and Supplementary Fig. 2A). Among the aged individuals, those cases with lung pathology presented a higher number of p16^INK4A^ and p21^CIP^ positive cells, reaching 3-fold average elevation, although this difference did not reach statistical significance (Fig. 2C). Moreover, we observed a significant decrease of cells negative for Lamin B1 with aging (Fig. 2B). Further analysis by IF revealed that cells from individuals with lung pathology displayed lower intensity of Lamin B1 compared to aged and young groups, respectively (Fig. 2D, E).

Trying to identify whether stem cells become senescent with physiological and pathological aging, we performed double IF with p16^INK4A^ and p63 markers, as well as p21^CIP^ and Lamin B1 with KRT5. We detected p16^INK4A^ and p21^CIP^ positive cells in the aged group but colocalization of p63 and p16^INK4A^ or KRT5 and p21^CIP^ in aged patients with lung pathology (Fig. 2F, G). In both cases, the percentage of double positive cells was below 10% relative to the number of basal p63 or KRT5 positive cells alone (Fig. 2H, I and Supplementary Fig. 2B, C). Additionally, the intensity of Lamin B1 staining was lower in KRT5 positive cells in the group of lung pathology cases (Fig. 2J). Our data suggest that resident cells in the conducting airway epithelium become senescent with age, affecting basal cells mostly in the case of pathological aging.

In the case of proliferation rate, no significant changes in Ki67 positive cells were found, although aged individuals showed a trend of higher Ki67 expression, especially in the case of aged patients with pulmonary diseases (Fig. 2A-C). We performed double IF with Ki67 and KRT5 markers to determine if lung basal cells were proliferative in the different groups of patients. We observed few cells with colocalization of Ki67 and KRT5 in conducting airways (Fig. 3A, B), suggesting a majority of non-proliferative basal cells in the different groups.

Finally, we measured the expression of keratin 14 (KRT14), considered a marker of lung repair/remodeling, with the majority of KRT5^+^ basal cells also expressing it during repair processes [2, 29]. Notably, IHC detected positive cells in rare cases of the young or aged group, whereas identified a low percentage of cells positive for KRT14 (≈5%) in some aged individuals with lung pathology (Fig. 2A-C). Indeed, the expression of KRT14 was mainly detected in the cases with widespread fibrosis and presence of dysplastic airway-like structures (Supplementary Fig. 3). However, in this region, we were able to detect few cells with colocalization of KRT14 and KRT5 (Fig. 3C). Similarly, the expression of the different markers was not affected when cases were divided by gender (Supplementary Fig. 4).

### SOX2^+^/p63^+^/KRT5^+^ cells increase with pathological aging at the exchange zone

Next, we moved to the exchange zone to study the same stem cell markers in the alveoli and/or dysplastic structures. The status of lung stem cells in this region was different compared to small airways. Remarkably, IHC and IF revealed that SOX2^+^, p63^+^ and KRT5^+^ cells were only expressed in some aged individuals (Fig. 4A, B and Supplementary Fig. 5A), being at significantly higher levels in those cases diagnosed with lung pathology (Fig. 4C, D). Notably, SOX2, p63 and KRT5 expression was mostly associated with dysplastic airway-like structures found in aged individuals with pathology and lung structural damage (Fig. 4A, B and Supplementary Fig. 5B). Additionally, IHC showed a similar number of SOX2^+^, p63^+^, and KRT5^+^ cells in aged cases with pathology who expressed an average of 14.3%, 11.8% and 22.1% positive cells, respectively, compared to 2.1%, 2% and 2.4% in aged individuals without disease and 0% in young individuals (Fig. 4D). Most cells were SOX2^+^, whereas the expression of p63 and KRT5 was restricted to cells located at the base of dysplastic airway-like structures where epithelium organization resembles the normal bronchial epithelium. On the other hand, the number of positive cells for the TTF-1 differentiation marker remained at the same level in aged *vs.* young individuals (Fig. 4A, C).

To further characterize which type of cells were trying to repopulate the damaged alveoli in the aged pathology group, we performed triple IF using SOX2, p63 and KRT5 markers. We only observed SOX2^+^/p63^+^/KRT5^+^ cells in aged individuals with pulmonary disease and dysplastic airway-like structures (Fig. 4B, E). Consistent with the lung remodeling attempt, we detected an increment of KRT14^+^ cells in cases with lung pathologies, who were the group that mostly expressed this marker in the exchange zone (Fig. 4F, Supplementary Fig. 5C). In line with this, KRT14^+^ cells colocalized with KRT5^+^ in dysplastic airway-like structures (Fig. 4F), which were mostly negative for the Ki67 proliferative marker (Supplementary Fig. 5D). Furthermore, we identified again two sets of cells in dysplastic structures: triple positive cells (SOX2^+^/p63^+^/KRT5^+^) and SOX2^+^ cells, which expressed KRT8 (Fig. 4G). All data together suggest the presence of a population of SOX2^+^/p63^+^/KRT5^+^ cells at dysplastic airway-like structures in aged individuals with severe lung pathology.

### Senescent cells increase with pathological aging at the exchange zone

We also studied the senescence and proliferation rate in the exchange zone. On the one hand, p16^INK4A^ and p21^CIP^ markers were only expressed in aged individuals and in higher proportion than in conducting airway epithelium (Fig. 5A-C *vs.* 2A-C). Among the aged groups, p16^INK4A+^ and p21^CIP+^ cells were more than 3 times more abundant in cases diagnosed with pulmonary diseases (28.5 and 12.1%) than in aged individuals without lung pathology (9.6 and 0.71%) (Fig. 5C). Moreover, among the cases with lung pathology the number of positive cells for p16^INK4A^ and p21^CIP^ was higher in the areas of dysplastic airway-like structures (Supplementary Fig. 5C). Supporting the enhanced accumulation of senescent cells in these cases, the number of cells negative for Lamin B1 was significantly decreased in the aged group (Fig. 5C), with the cases of lung pathology displaying the lowest intensity (Fig. 5D, E). In contrast, no differences in cell proliferation were detected since Ki67^+^ cells remained largely unchanged in the exchange zone for the three groups of patients (Fig. 5C).

To characterize whether the population of stem cells undergo senescence, double IF of p16^INK4A^ and p63, as well as p21^CIP^ and Lamin B1 with KRT5, were performed. Data from this assay revealed colocalization of senescence p16^INK4A^ and p21^CIP^ markers with p63 and KRT5 basal cell markers only in aged patients with pulmonary disease (Fig. 5F, G and Supplemntart Fig. 6A-C). Remarkably, we observed a marked increment of senescent basal cells in the exchange zone (double p63^+^/p16^INK4A+^ ≈40% and KRT5^+^/p21^CIP+^ ≈15%) compared to conducting airway epithelium (≈12.5 and 4%, respectively) (Fig. 5H, I vs. 2H, I). Similarly, we quantified the number of cells with low staining of Lamin B1 (intensity below average measured in aged cases), finding low number in young cases (≈10%) and the highest proportion of these cells in lung pathology cases (≈75%) (Fig. 5J). Among them, over 85% of KRT5 positive cells displayed low Lamin B1 staining.

Concerning proliferation, no differences in Ki67 positive cells were observed between the three groups (Fig. 6A, B). Moreover, the same trend was observed for Ki67^+^/KRT5^+^ cells, which were only observed in aged patients with lung pathology (Fig. 6C). Notably, the number of proliferative basal cells measured as Ki67^+^/KRT5^+^ was ≈5% relative to the total KRT5^+^ population (Fig. 6D), lower percentage than the senescent population. In this line, triple p63/p16^INK4A^/Ki67 IF confirmed this result and revealed that senescence and proliferation markers are mutually exclusive (Fig. 6E). Moreover, the ratio of p63/p16^INK4A^ cells was 6 times higher than p63/Ki67 proliferative cells (Fig. 6F). These results suggest that SOX2^+^/p63^+^/KRT5^+^ lung basal stem cells try to repair damaged tissue in the exchange zone where they likely undergo senescence.

## DISCUSSION

The basic biological function of lungs is performing gas exchange of oxygen-for-carbon dioxide in alveoli, which requires the correct activation of homeostasis mechanisms to maintain normal lung physiology and structure. With aging, progressive functional and structural deterioration of lung occurs. However, critical cellular and molecular mechanisms underlying the physiopathology of aging such as stem cell activity and senescence in human lung have not been explored so far.

The source of cells repopulating damaged alveolar regions has been characterized in mice. The main recognized mechanism to repair severe lung injuries consists of colonization of damaged alveoli by basal cells (p63^+^/KRT5^+^) and specialized epithelial SOX2^+^ progenitor cell populations coming from the airway epithelium [3-5, 30]. On the other hand, alveolar repair can be driven by alveolar type II cells, which differentiate to transitional stem cell states that precede the regeneration of alveolar type I cells. In detail, these intermediate progenitors are characterized by the expression of KRT8, enrichment of tumor protein p53 (TP53) and hypoxia inducible factor 1 subunit alpha (HIF1A) signaling as well as a senescence transcriptional signature [31-33]. These findings in mice extend to human lung diseases such as IPF, as accumulation of KRT8^+^ transitional states was observed in fibrotic regions [31-33]. Interestingly, these intermediate cell states also express p63 and keratin 17 (KRT17) [33]. Human alveolar type II cells can also transdifferentiate into metaplastic KRT5^+^ basal cells in response to an IPF pro-inflammatory context [34].

In human lung airways, we characterized SOX2, p63 and KRT5 expression and describe two different cell populations: One characterized by colocalization of SOX2, p63 and KRT5 markers, coincident with basal cells, and a second population of differentiated cells positive for SOX2, including ciliated and secretory cells. Surprisingly, the number of p63^+^ and KRT5^+^ basal cells did not vary with physiological and pathological aging compared to young samples. These results differ from mice where there is a decline in KRT5^+^/p63^+^ basal cells in the tracheal epithelium with age [10, 12, 13]. Mice have significant structural and functional differences with human lungs, especially in the case of distal airways [1], which might explain the differences between mouse and human samples. It can be noted as well that different stem cells, mainly hematopoietic progenitors, display impaired function and not decline in number with age [35, 36]. Additionally, we observed a reduced number of SOX2 positive cells and SOX2 expression in conducting airways with aging, especially in aged individuals with pulmonary disease. This might suggest SOX2^+^ cells could retain stem cell activity and be involved in differentiation and/or dedifferentiation as an alternative source of lung basal cells. Consistent with this, we detected co-staining of SOX2 with KRT8 expression, which could indicate the presence of intermediate progenitors involved in lung regeneration. Additional SOX2^+^ populations would be club cells, putative stem cells able to dedifferentiate into KRT5^+^ basal cells in the mouse airway epithelium [37, 38]. In this line, SOX2^+^/SCGB1A1^-^/KRT5^-^ proximal airway stem cells give rise to KRT5^+^/p63^+^ pods that repopulate mouse small airways and alveoli after H1N1 influenza virus infection [4, 6]. This hypothesis is also supported by a recent work that identified a population of pre-existing, rare SOX2^+^/SCGB1A1^+^ stem cells (5%) different from basal cells (p63^-^) that mobilize after major injury to repair alveolar units in mice [30]. They showed that dedifferentiation of quiescent, Sox2^+^ club-like mature cells constitutes a major pathway of lung regeneration giving rise to alveolar lineages *in vivo*; although these cells have impaired self-renewal and features of senescence that limit complete alveolar repair [30]. Finally, the existence of different potential stem cell populations in lung regeneration may imply hierarchical repair responses in humans according to the type and intensity of injury as previously described in mice [4, 39]. Together with this, we showed an increment in p16^INK4A+^, p21^CIP+^ and Lamin B1 low senescent cells in the respiratory epithelium, especially in the group of aged patients with lung pathology. In this group, we detected double p63/p16^INK4A^ positive cells, but not in the other two sets of patients, as well as double KRT5/p21^CIP^ and basal cells with Lamin B1 low staining. This led us to speculate that human aged basal cells are prone to senescence and that would compromise their regeneration capacity.

In the exchange zone, we detected SOX2^+^, p63^+^, and KRT5^+^ cells in aged individuals diagnosed with lung disease. In detail, we observed dysplastic airway-like structures that substitute normal alveolar units in those aged patients with lung structural injury, who have severe and/or long-term damage. These structures express stem cell and differentiation markers and present colocalization of p63 and KRT5 in basal cells, as previously reported in mouse models [30, 40]. Therefore, our results suggest that alveolar damage with aging promotes p63^+^/KRT5^+^ stem cell mobilization, trying to regenerate damaged structures as supported by the expression of KRT14 regeneration marker as well as colocalization of KRT5 and Ki67 proliferation marker. It should be taken into account that our study is observational and lacking lineage-tracing analysis that could confirm basal cell mobilization. In line with our result of colocalization of KRT5 basal marker and KRT14 in dysplastic airway-like structures, an additional study also detected it in patients with IPF [41]. Moreover, these dysplastic regions also show reduced expression of SOX2 and high levels of senescence markers p16^INK4A^ and p21^CIP^, as well as low Lamin B1, which points out that the regeneration attempt is not successful when the damage is too high and extensive as observed in patients with lung structural pathology. This is due to basal SOX2^+^/p63^+^/KRT5^+^ stem cells becoming probably senescent, thus preventing them to differentiate and repopulate the damaged structures, which entails the first description in humans of this process. This idea is supported by studies in mice [42], the mutual exclusive staining of proliferation and senescence markers, and the higher ratio of senescent compared to proliferative stem cells. Therefore, the alterations in stem cell activity and senescence accumulation might constitute the molecular basis of fibrosis deposition and loss of normal alveolar structure observed in aged patients with pathology.

## Supplementary Materials

The Supplementary data can be found online at: www.aginganddisease.org/EN/10.14336/AD.2022.1128.
