# Supplementary material for: KRT5+/p63+ Stem Cells Undergo Senescence in the Human Lung with Pathological Aging
Source: Aging Dis. 2023 Jun 1;14(3):1013–27. doi: 10.14336/AD.2022.1128 (PMC10187699; doi:10.14336/AD.2022.1128)
Supplement: Supplementary file 1 — The Supplementary data can be found online at: www.aginganddisease.org/EN/10.14336/AD.2022.1128. [file AD-14-3-1013-s.pdf]

SUPPLEMENTARY DATA

KRT5$^+$/p63$^+$ Stem Cells Undergo Senescence in the Human Lung with Pathological Aging

Manuel Moreno-Valladares[1,2,3#], Veronica Moncho-Amor[1,3#], Tulio M Silva[2], Juan P Garcés[2], María Álvarez-Satta[1,3], Ander Matheu[1,3,4*]

© 2022. Moreno-Valladares M, et al. Published online at http://www.aginganddisease.org/EN/10.14336/AD.2022.1128

SUPPLEMENTARY DATA

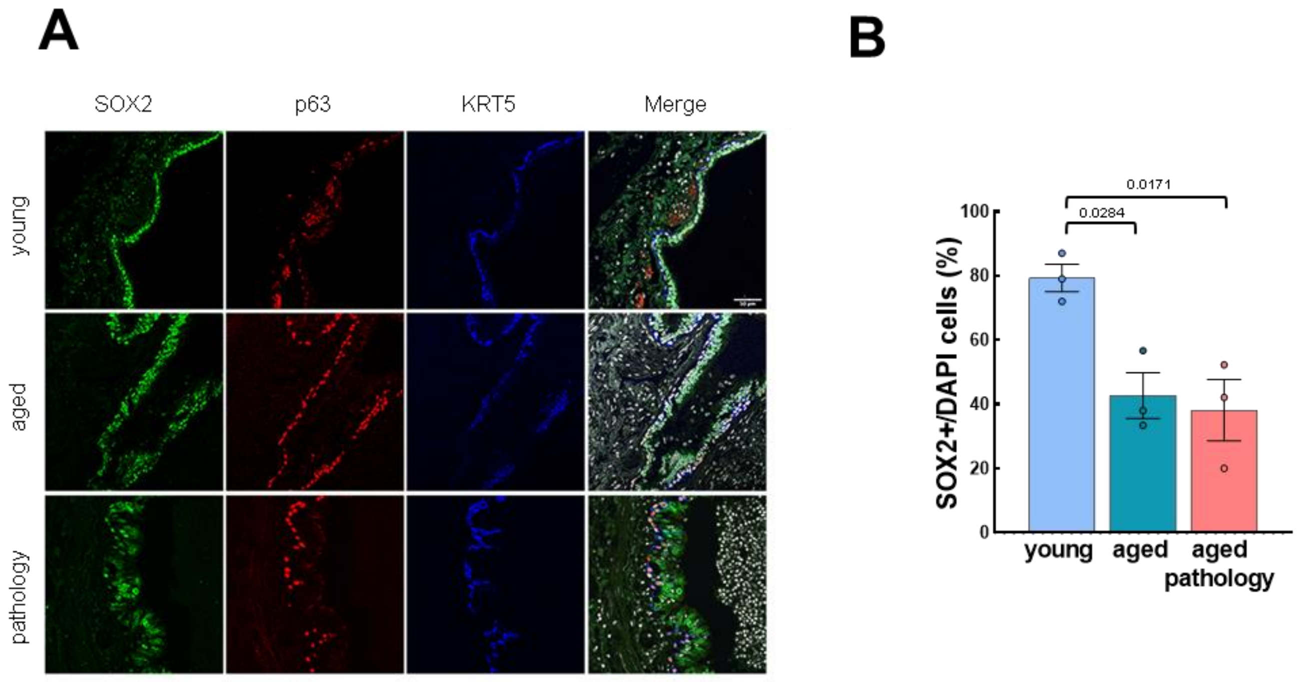

Supplementary Figure 1. SOX2, p63 and KRT5 staining in conducting airway epithelium. (A) Representative IF staining of SOX2, p63, KRT5 and merged pictures with DAPI counterstaining in the three groups of individuals (rows). Scale bar: 50 µm. **(B)** Quantification of $SOX2^{+}$ cells relative to total DAPI cells. Data represent average ± SEM (n=3 individuals per group). p-value when statistical significance was reached, is shown.

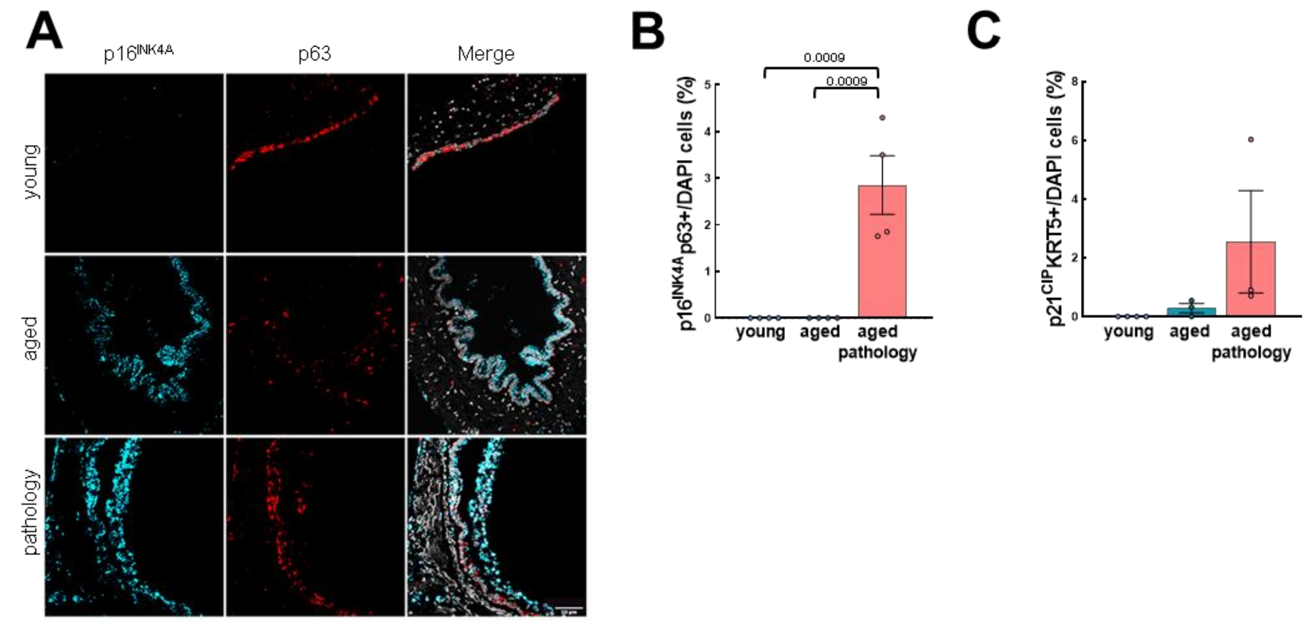

Supplementary Figure 2. Senescence markers in conducting airway epithelium. (A) Representative IF staining of $p16^{INK4A}$, p63 and merged pictures with DAPI counterstaining in the three in the three groups of individuals (rows). Scale bar: 50 µm **(B-C)** Quantification of double positive cells for $p16^{INK4A}$/p63 and $p21^{CIP}$/KRT5 relative to total DAPI cells (n>3). Data represent average ± SEM. p-value when statistical significance was reached, is shown.

© 2022. Moreno-Valladares M, et al. Published online at http://www.aginganddisease.org/EN/10.14336/AD.2022.1128

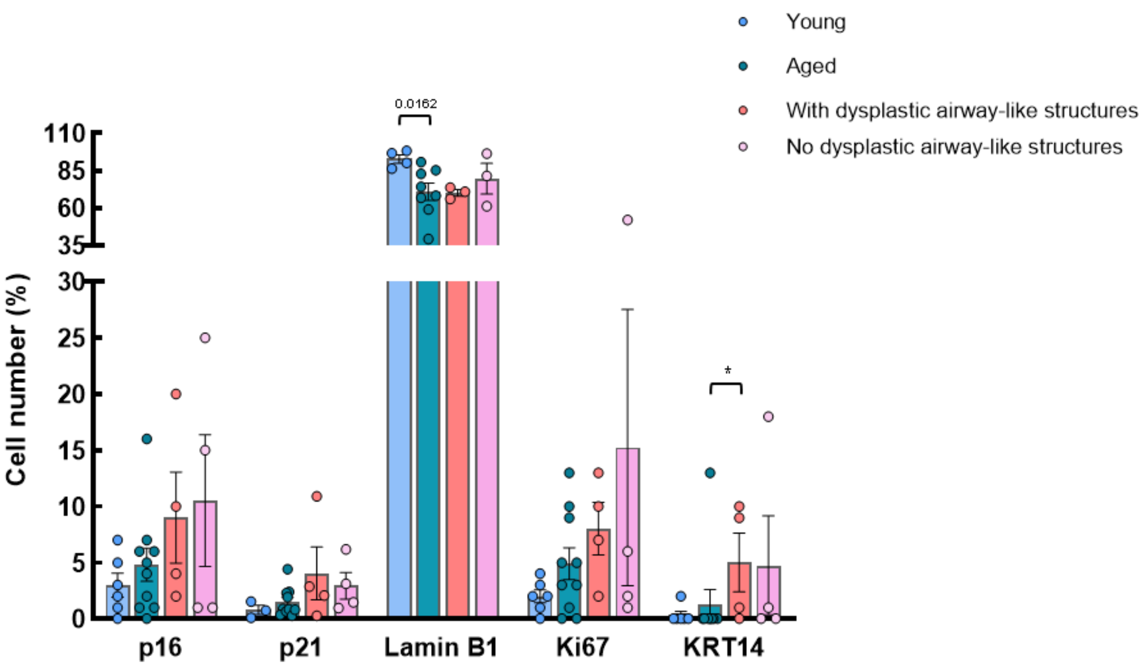

Supplementary Figure 3. Senescence markers and KRT14 in conducting airway epithelium. Quantification of positive cells for indicated proteins by IHC in the different groups and divided by severity of pathology (young: n≥3; aged with no lung pathology: n≥8; pathology aged with dysplastic airway-like structures: n≥3; without dysplastic airway-like structures: n≥3). Data represent average ± SEM. p-value when statistical significance was reached, is shown.

© 2022. Moreno-Valladares M, et al. Published online at http://www.aginganddisease.org/EN/10.14336/AD.2022.1128

SUPPLEMENTARY DATA

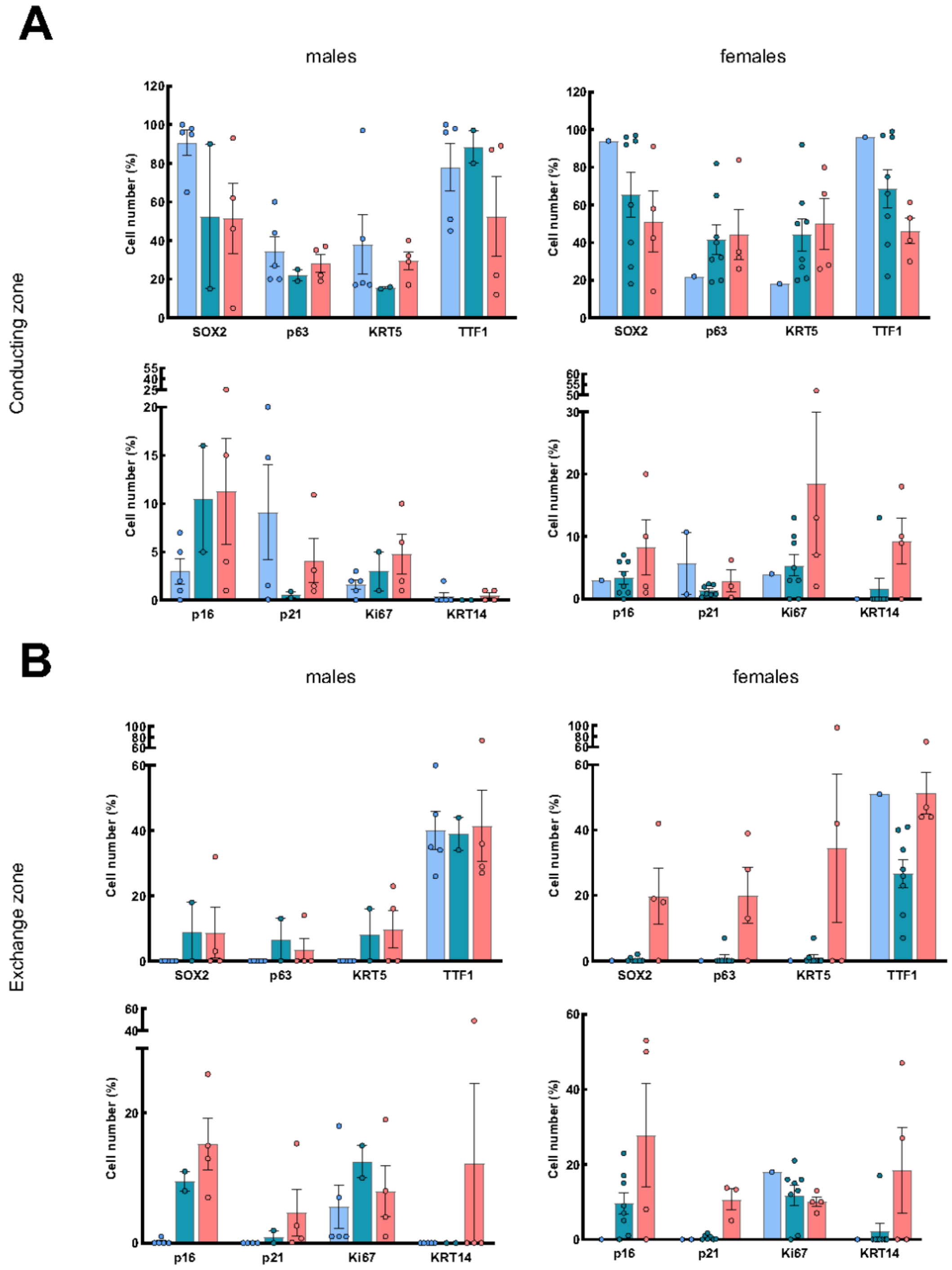

Supplementary Figure 4. Expression of different markers divided by gender. (A) Quantification of cells positive for indicated markers divided by males and females in conducting zone. **(B)** Quantification of cells positive for indicated markers divided by males and females in exchange zone.

© 2022. Moreno-Valladares M, et al. Published online at http://www.aginganddisease.org/EN/10.14336/AD.2022.1128

SUPPLEMENTARY DATA

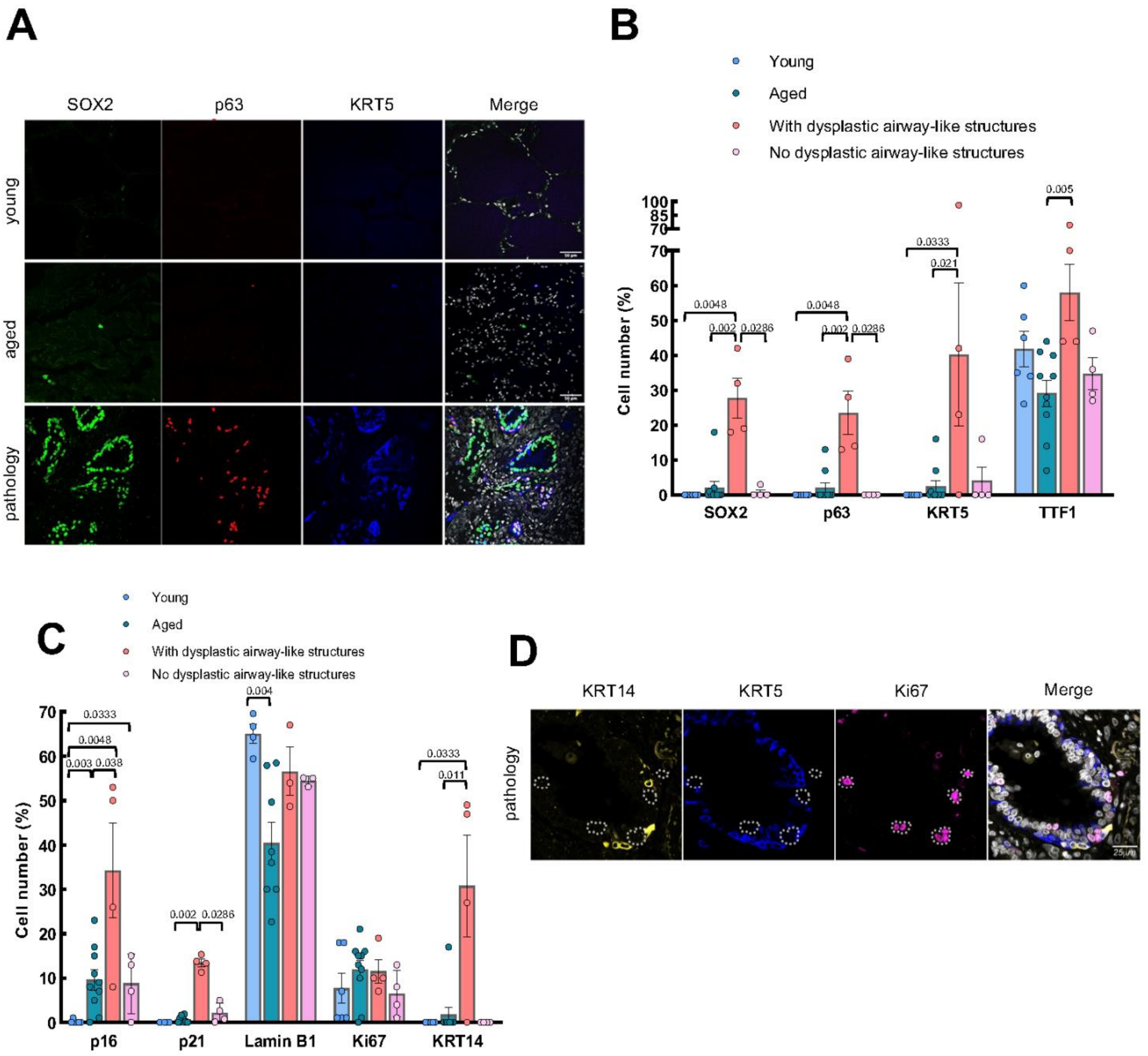

Supplementary Figure 5. SOX2, p63 and KRT5 and KRT14 expression in the exchange zone. (A) Representative IF staining of SOX2, p63, KRT5 and merged pictures with DAPI counterstaining in the three groups of individuals (rows). Scale bar: 50 µm. **(B)** Quantification of cells positive for SOX2, p63, KRT5 and TTF-1 markers by IHC (young: n≥3; aged with no lung pathology: n≥8; pathology aged with dysplastic airway-like structures: n≥3; without dysplastic airway-like structures: n≥3). **(C)** Quantification of cells positive for p16^{INK4a}, p21^{CIP}, Lamin B1, Ki67 and KRT14 markers by IHC (young: n≥3; aged with no lung pathology: n≥8; pathology aged with dysplastic airway-like structures: n≥3; without dysplastic airway-like structures: n≥3). Data are shown as percentage of marked cells relative to total nuclei. **(D)** Representative IF staining of KRT5, KRT14, Ki67 and merged pictures with DAPI counterstaining in the aged pathology group. Data represent average ± SEM. p-value when statistical significance was reached, is shown.

© 2022. Moreno-Valladares M, et al. Published online at http://www.aginganddisease.org/EN/10.14336/AD.2022.1128

SUPPLEMENTARY DATA

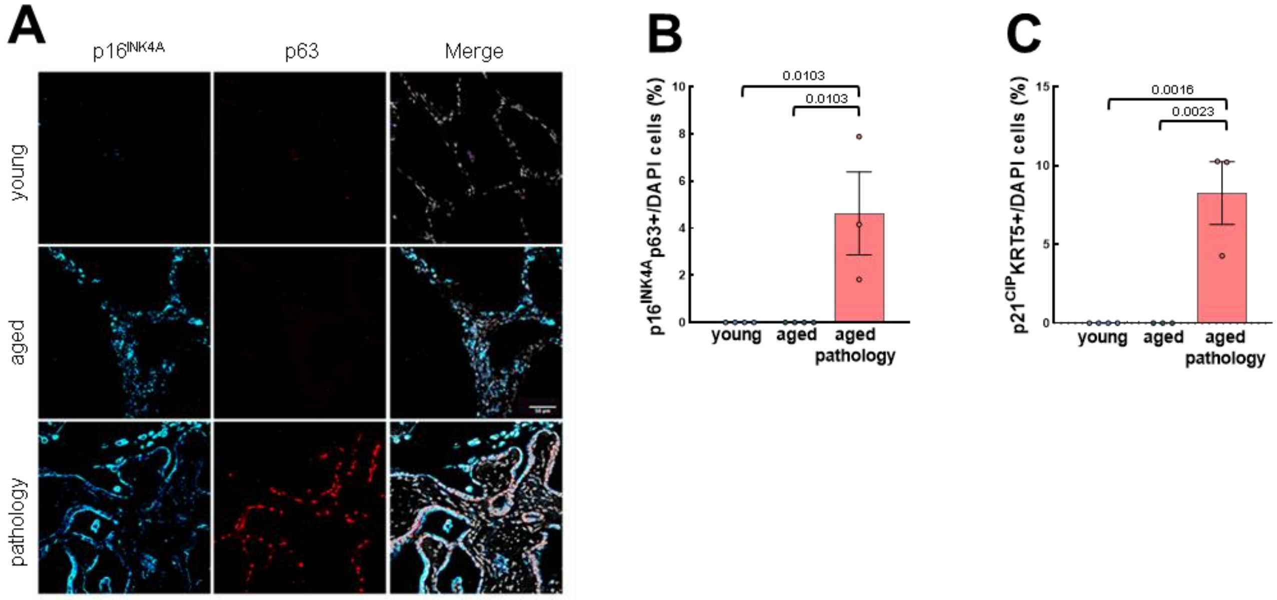

Supplementary Figure 6. Senescence markers in the exchange zone. (A) Representative IF staining of p16^{INK4A}, p63 and merged pictures with DAPI counterstaining in the three groups of individuals (rows). Scale bar: 50 µm. **(B,C)** Quantification of double positive cells for p16^{INK4A} and p63 or p21^{CIP} and KRT5 markers relative to total nuclei in the three groups of individuals (young: n=3; aged and aged pathology: n=4). Data represent average ± SEM. p-value when statistical significance was reached, is shown.

© 2022. Moreno-Valladares M, et al. Published online at http://www.aginganddisease.org/EN/10.14336/AD.2022.1128
